# Performance of the SRK/T formula using A-Scan ultrasound biometry after phacoemulsification in eyes with short and long axial lengths

**DOI:** 10.1186/s12886-016-0271-8

**Published:** 2016-07-08

**Authors:** Yunus Karabela, Mustafa Eliacik, Faruk Kaya

**Affiliations:** Department of Ophthalmology, Istanbul Medipol University, Esenler Hospital, Birlik Mah., Bahceler Cad., Number 5, Esenler, Istanbul, 34230 Turkey; Department of Ophthalmology, School of Medicine, Istanbul Medipol University, Kadıkoy Medipol Hospital, Lambaci Sokak Number 1, Kosuyolu, Kadikoy, Istanbul, 34718 Turkey

**Keywords:** IOL power calculation, SRK/T, Refractive outcome, Short axial length, Long axial length, Cataract surgery

## Abstract

**Background:**

The SRK/T formula is one of the third generation IOL calculation formulas. The purpose of this study was to evaluate the performance of the SRK/T formula in predicting a target refraction ±1.0D in short and long eyes using ultrasound biometry after phacoemulsification.

**Methods:**

The present study was a retrospective analysis, which included 38 eyes with an AL < 22.0 mm (short AL), and 62 eyes ≥24.6 mm (long AL) that underwent uncomplicated phacoemulsification. Preoperative AL was measured by ultrasound biometry and SRK/T formula was used for IOL calculation. Three different IOLs were implanted in the capsular bag. The prediction error was defined as the difference between the achieved postoperative refraction, and attempted predicted target refraction. Statistical analysis was performed with SPSS V21.

**Results:**

In short ALs, the mean age was 65.13 ± 9.49 year, the mean AL was 21.55 ± 0.45 mm, the mean K1 and K2 were 45.76 ± 1.77D and 46.09 ± 1.61D, the mean IOL power was 23.96 ± 1.92D, the mean attempted (predicted) value was 0.07 ± 0.26D, the mean achieved value was 0.07 ± 0.63 D, the mean PE was 0.01 ± 0.60D, and the MAE was 0.51 ± 0.31D. A significant positive relationship with AL and K1, K2, IOL power and a strong negative relationship with PE and achieved postoperative was found. In long ALs, the mean age was 64.05 ± 7.31 year, the mean AL was 25.77 ± 1.64 mm, the mean K1 and K2 were 42.20 ± 1.57D and 42.17 ± 1.68D, the mean IOL power was 15.79 ± 5.17D, the mean attempted value was −0.434 ± 0.315D, the mean achieved value was −0.42 ± 0.96D, the mean PE was −0.004 ± 0.93D, the MAE was 0.68 ± 0.62D. A significant positive relationship with AL and K1, K2 and a significant positive relationship with PE and achieved value, otherwise a negative relationship with AL and IOL power was found. There was a little tendency towards hyperopic for short ALs and myopic for long ALs. The majority of eyes (94.74 %) for short ALs and (70.97 %) for long ALs were within ±1 D of the predicted refractive error. No significant relationship with PE and IOL types, AL, K1, K2, IOL power, and attempted value, besides with MAE and AL, K1, K2, age, attempted, achieved value were found in both groups.

**Conclusion:**

The SRK/T formula performs well and shows good predictability in eyes with short and long axial lengths.

## Background

Cataract surgery is the most frequently performed ophthalmic surgery in the world. With the advanced technology and improvement in surgical techniques, final refractive outcomes and patient satisfaction are essential for determining the success of this procedure [1]. To achieve optimum outcomes, preoperative biometry must be accurate and an accurate IOL power (IOLp) formula must be used [2].

Intraocular lens (IOL) power calculation formulas have been evolving since 1949 when Harold Ridley implanted the first IOL into a human eye [3]. Various theoretical and regression formula are available for calculation of IOL power. Holladay 1 [4], Hoffer Q [5], and SRK-T [6] are known as third generation formulas and Holladay 2 [7], Haigis [8], Olsen [9] as fourth or newer generation formulas. Although third and fourth generation formulas are well accurate in eyes with average axial length, there is no general consensus as to which formula for IOL measurement is the most accurate in short or long eyes [1, 4–9].

Retzlaff JA, Sanders DR, and Kraff MC developed the SRK/T formula in 1990. The SRK/T (T for theoretical) is a formula, representing a combination of linear regression method with a theoretical eye model [6].

Ultrasound (US) biometry (A-Scan) and partial coherence interferometer (PCI)-based devices are the most commonly used methods for determining IOL power [10]. Previous comparisons of ultrasound biometry and optical biometry were reported equal or better results with optical biometry. However, ultrasound biometry remains the preferred method of measuring the axial length in the most practices, especially in developing countries or dense ocular media or inadequate measurements of PCI-based device [10–16].

The purpose of this study is to evaluate the performance of the SRK/T formula using contact ultrasound biometry in predicting a target postoperative refraction ±1.0 D in eyes with short and long AL after phacoemulsification and foldable lens implantation.

## Methods

The records of all patients who had uncomplicated phacoemulsification with implantation of foldable IOL in the capsular bag between 2006 and 2012 at the Nisa Hospital, Istanbul, Turkey, were retrospectively reviewed. Patients were divided into 2 groups based on AL<22.00 mm (Group1, short ALs), and ≥24.6 mm (Group2, long ALs). Phacoemulsification was performed using Sovereign Compact Cataract Extraction System (Abbott Medical Optics Inc., Illionis, USA) and the foldable IOL was implanted in the capsular bag, through a 3.0–3.5 mm clear corneal incision by a single surgeon (YK). Three types of IOLs were used in this present study; Softec 1(Lenstec Inc., St. Petersburg, FL, USA), DrSchmidt (HumanOptics AG, Erlangen, Germany), Acriva (VSY, Istanbul,Turkey). Patients with intraoperative and postoperative complications, pre-existing astigmatism > 2.5 D, history of previous ocular surgery or injury, and presence of associated ocular pathologies, monocular patients, patients in whom IOL power was calculated with other formulas, patients with incomplete pre or postoperative data were excluded.

Preoperatively, all patients underwent a full ophthalmological examination including uncorrected and best-corrected Snellen visual acuity, intraocular pressure (IOP), slit-lamp and fundus examination, biometry for IOL power calculation including keratometry, and AL measurements. Refraction and keratometry were carried out by using the autokerato-refractometer (Topcon KR 8000, Japan). The axial length was measured by the contact method using A-Scan ultrasonic biometer (EZ AB5500+ A-Scan/B-Scan; Sonomed Inc., Lake Success, NY, USA). The SRK/T formula was chosen to predict the IOL power. The manufacturers’ recommended A-constants were used for the IOL type. The surgeon’s goal in IOL power selection was a lens power that would yield a postoperative refraction ±1.0D accurate. All patients were evaluated on postoperative days 1, 7 and 30. The final refraction carried out with the autokerato-refractometer at 30 days postoperatively and confirmed by subjective refraction. All records of the refraction were converted into a spherical equivalent value, which was taken as the refractive outcome. Postoperative refractive prediction errors, mean PE and MAE were calculated for all patients.

The retrospective study was approved by the Ethics Committee of the Istanbul Medipol University, and was conducted in accordance with the tenets of the Declaration of Helsinki by obtaining written informed consent from all patients.

### Statistical analysis

Statistical analysis was conducted using SPSS software (21.0, SPSS Inc., Chicago, IL, USA). Values were recorded as mean ± SD (standard deviation). A test of the normality of the data distribution was performed using the Shapiro-Wilk tests. The correlation between prediction error(PE) and AL, K1, K2, IOL power and age of the patient was made using the Pearson’s and Sperman’s rank correlation coefficient depending on the normality of the data. In all cases, a *p*-value less than 0.05 were considered statistically significant. Paired Samples *t* test was used for difference between attempted and achieved spherival equivalent in both groups. Additionally, a comparison between the groups of different IOL types was made using the one-way ANOVA in both groups.

## Results

### In group 1 (Short ALs)

A total 38 eyes with short ALs from 29 patients were included in this group. The mean age of patients was 65.13 ± 9.49 year (range 41 to 80), the mean AL was 21.55 ± 0.45 mm (range 20.05 to 21.99), the mean K1 was 45.76 ± 1.77 D (range 42.00 to 49.75), the mean K2 was 46.09 ± 1.61 D (range 41.87 to 48.25), the mean IOL power was 23.96 ± 1.92 D (range 21 to 30), the mean attempted preoperative spherical equivalent (attempted SE) was 0.07 ± 0.26 D (range −0.26 to 0.89), the mean achieved spherical equivalent (achieved SE) was 0.07 ± 0.63 D (range −1.0 to 1.50), the mean prediction error(PE) was 0.01 ± 0.60D (range −1.015 to 1.060), the mean absolute error(MAE) was 0.51 ± 0.31D (range 0.02 to 1.060).

Pre-operative and demographic parameters are summarized in Table 1 and distribution of the prediction error (difference between attempted and achieved spherical equivalent) in eyes with short AL is shown in Table 2.

**Table 1:** Preoperative and demographic parameters in eyes with short and long AL

	Short Eyes			Long Eyes		
	Range	Mean	SD	Range	Mean	SD
Age(year)	41–80	65.13	9.49	38–80	64.05	7.31
Axial length(mm)	20.05–21.99	21.55	0.45	24.60–32.90	25.77	1.64
Keratometry K_1_(D)	42.00–49.75	45.76	1.77	39.25–45.25	42.20	1.57
Keratometry K_2_(D)	41.87–48.25	46.09	1.61	39.62–46.00	42.17	1.68
IOL power(D)	21–30	23.96	1.92	−5.00-(20.50)	15.79	5.17
Gender	6 males (20.69 %) + 23 females (79.31 %)			33males(73.33 %) + 12 females(26.67 %)		
Eye	21 right eyes (55,3 %) +17 left eyes (44.7 %)			30 right eyes(48.4 %) + 32 left eyes (51.6 %)

**Table 2:** Distribution of the prediction error (difference between attempted and achieved spherical equivalent) in eyes with short and long AL using SRK/T formula and ultrasound biometry

Range of SE(D)	Short Eyes		Long Eyes	
	n	%	n	%
Within ±0.25 D	10	26.32	14	22.58
Within ±0.50 D	22	57.89	31	50.00
Within ±1.0 D	36	94.74	44	70.97
> + 1.0 D (more hyperopic than predicted)	1	2.63	3	4.84
*≥ + 2.0*	0	0.00	2	3.23
<-1.0 D (more myopic than predicted)	1	2.63	18	29.03
≤ − 2.0 D	0	0.00	3	4.84

*SE* spherical equivalent, *n* number of operated eyes

**Fig. 1:**
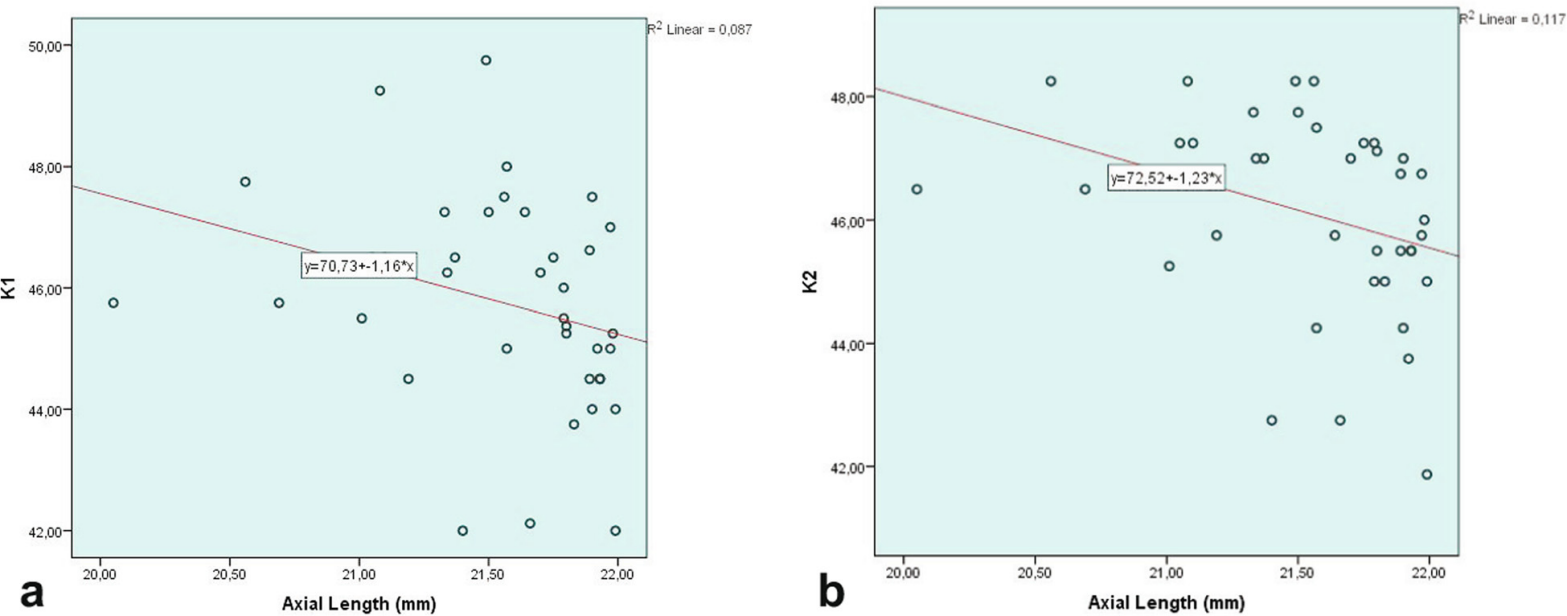
Scatter plot of AL versus K1 (**a**) and K2 (**b**) in eyes with short ALs

**Fig. 2:**
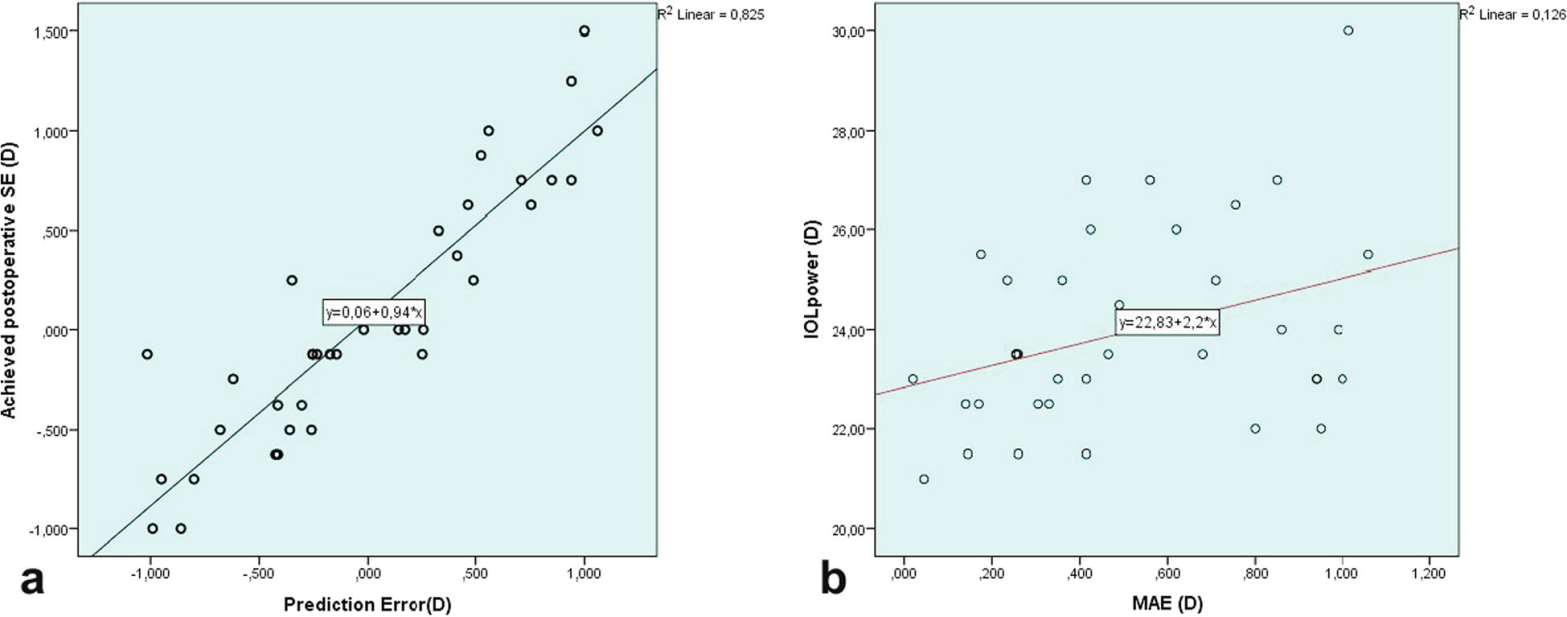
Scatter plot of prediction error versus achieved SE (**a**) and MAE versus IOL power (**b**) in eyes with short ALs

A statistically significant negative correlation was observed between AL and K1, K2, IOL power (*r* = −0.442, *p* = 0.05; *r* = −0.461, *p* = 0.04; *r* = −0.402, *p* = 0.012, respectively) (Fig. 1). A statistically significant positive correlation was found between MAE and IOL power*(r* = *0.355, p* = *0.029)* (Fig. 2). However, there was no significant correlation between MAE and AL, K1, K2, attempted SE or achieved SE. A statistically significant negative correlation was found between the mean PE and achieved SE (*r = −0.908, p = 0.00*) (Fig. 2).

No statistically significant relationship was found between mean PE and the other parameters. A weak positive, but statistically insignificant linear relation was observed between attempted preoperative SE and achieved postoperative SE(*r = 0.289; p = 0.078 > 0.05*). There was no statistically significant difference between attempted preoperative SE and achieved postoperative SE (*Paired samples t test; t (37) = −0.035, p = 0.972 > p = 0.05*)

In the present study, three types of IOL were used in short eyes. The Softec 1 was used in 25 eyes (65.8 %), the Dr Schmidt in 8 eyes (21.0 %), and the Acriva IOL in 5 eyes (13.2 %). There was no relation-ship was detected between the PE and and the type of IOL (*p = 0.631; p > 0. 05*) in short eyes.

### In group 2 (Long ALs)

There were 62 eyes of 45 patients (33 males, and 12 females). The mean age was 64.05 ± 7.31 year (range 38 to 80), the mean AL was 25.77 ± 1.64 mm (range 24.60 to 32.90 D), the mean K1 was 42.20 ± 1.57 D (range; 39.25 to 45.25 D), the mean K2 was 42.17 ± 1.68 D (range 39.62 to 46.00 D), the mean IOL power was 15.79 ± 5.17D (range −5,00 to 20.50 D), the mean attempted preoperative predictive spherical equivalent was −0.434 ± 0.315 D (range −1.00 to 0.54 D), the achieved postoperative spherical equivalent was −0.42 ± 0.96 D (range −2.62 to 2.75 D), the mean PE was −0.004 ± 0.93 D (range −1.83 to 3.55 D), the MAE was 0.68 ± 0.62D (range 0.005 to 3.55D).

### Pre-operative and demographic parameters are shown in Table 1 and distribution of the prediction error in eyes with long AL is shown in Table 2

The Softec 1 IOL was used in 48 eyes (77. 4 %), the Dr Schmidt in 10 eyes (16.1 %), and the Acriva IOL was in 4 eyes (6.5 %)

**Fig. 3:**
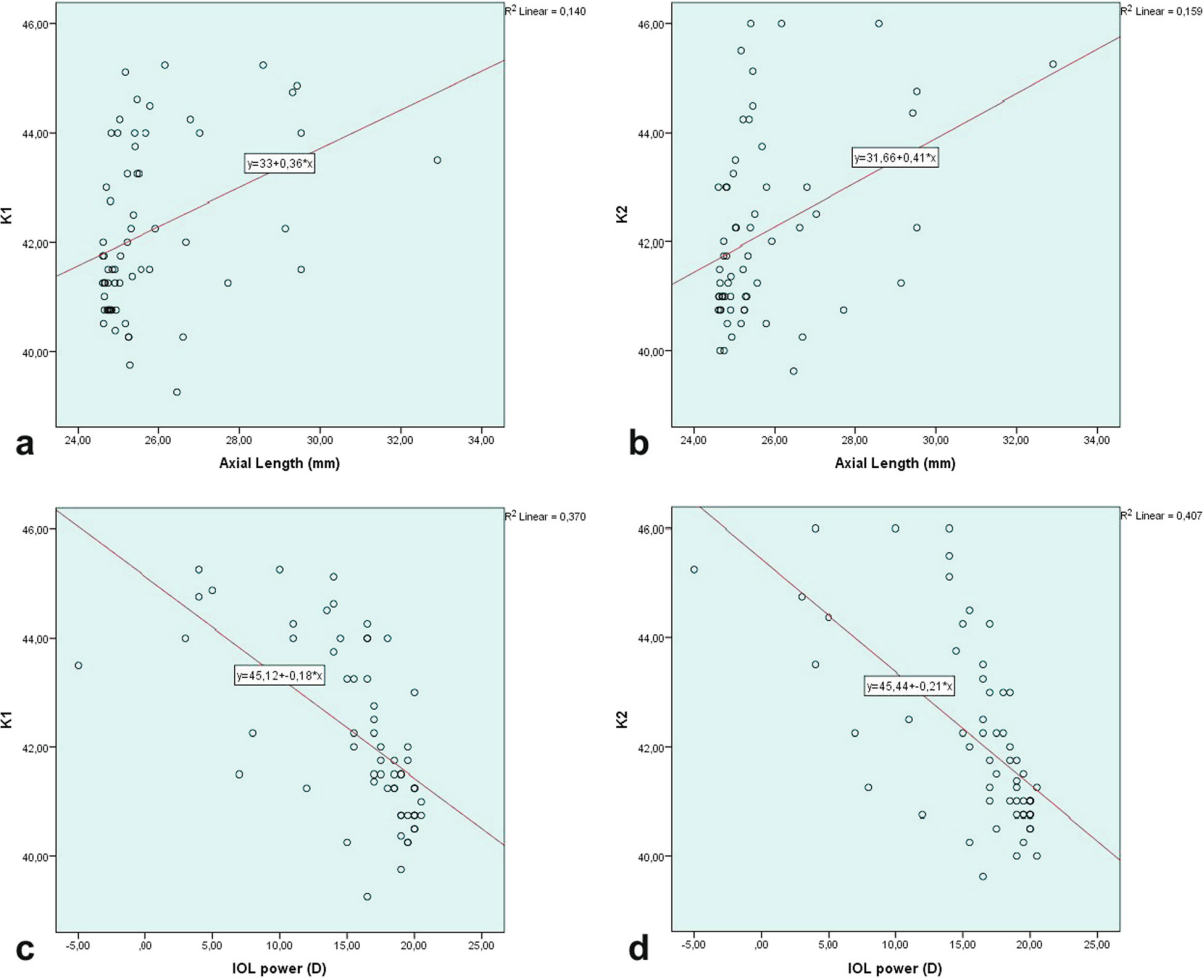
Scatter plot of AL versus K1 (**a**), K2 (**b**), and IOL power versus K1 (**c**), K2 (**d**) in eyes with long ALs

**Fig. 4:**
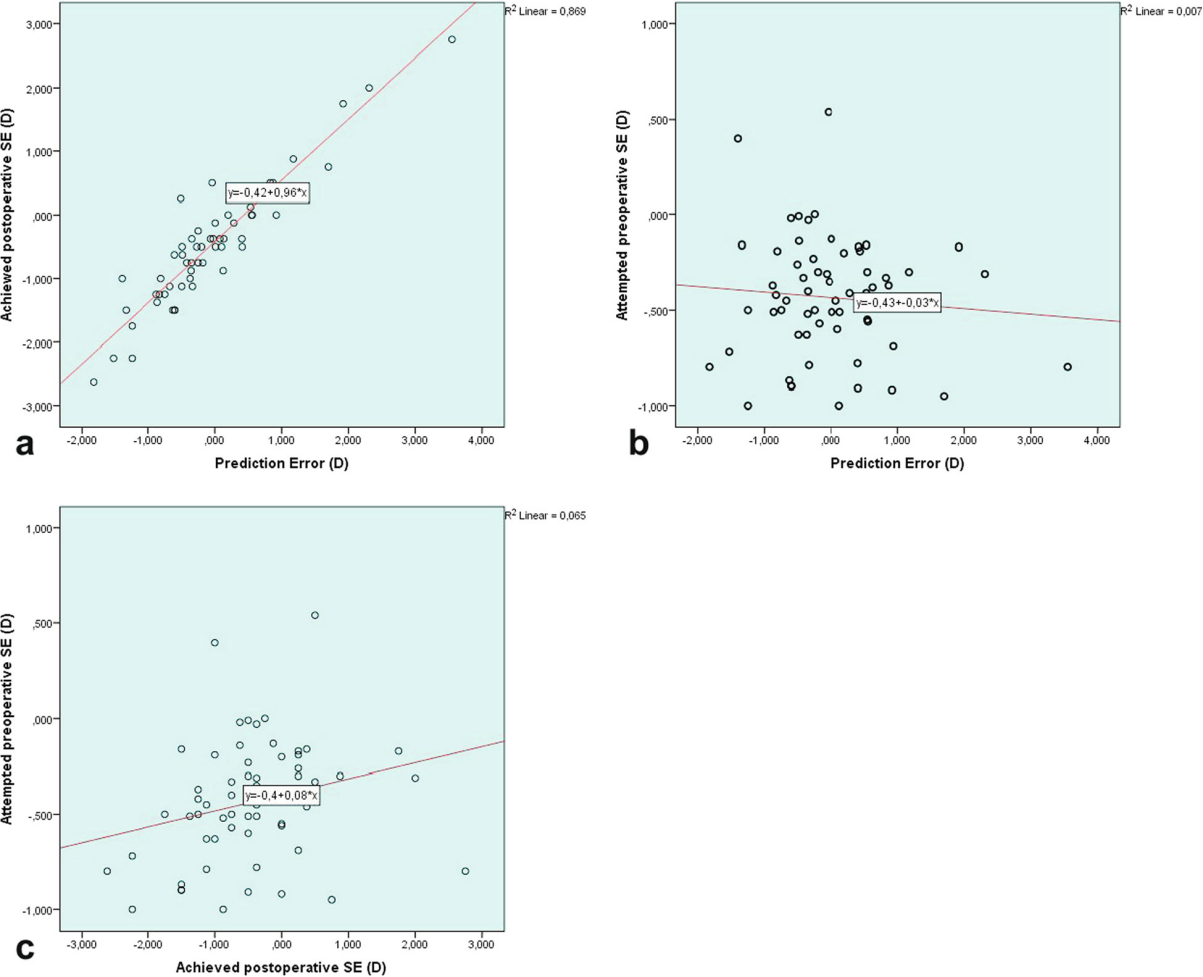
Scatter plot of prediction error versus achieved SE (**a**), attempted SE (**b**) in eyes with long ALs and achieved SE versus attempted SE (**c**)

In present study, there was a statistically significant positive relationship between AL and K1, K2 (*r = 0.432, p = 0.00 and r = 0.404, p = 0.001; respectively*) (Fig. 3). A negative statistically significant strong relationship was found between AL and IOL power (*r = −0.867 p = 0.00*)*.* No relationship was found between prediction and AL, K1, K2, IOLp, age. In addition, there was a strong positive correlation between PE and achieved postoperative SE(*r = 0.923, p = 0.00*) and no correlation with attempted preoperative SE (Fig. 4). There was a weak positive significant linear correlation between attempted preoperative SE and achieved postoperative SE(*r = 0.255; p = 0.045*) (Fig. 4). However, there was no significant difference in the values for attempted preoperative SE and achieved postoperative SE (*Paired samples t test; t (61) = −0.105, p = 0.917 > p = 0.05*). A strong negative relationship was found between IOL power and K1 and K2 (*r = −0.710, p = 0.00; r = −0.703, p = 0.00 respectively*) (Fig. 3)

No relation-ship was detected between the PE and the type of IOL (*p = 0.501; p > 0. 05*)

## Discussion

The SRK T formula is a third generation formula, described in 1990 by John Retzlaff, Kraff and Sanders [6]. This formula combines the benefits of both the theoretical and regression formula and uses the A-constant to calculate the ACD, using the retinal thickness and corneal refractive index. The ACD constant for SRK-T may be supplied by the manufacturer or may be calculated from the SRK-II [1, 3–6, 10–14, 16].

In the present retrospective study, we assessed the performance of the SRK/T formula using ultrasonic biometry in eyes with short and long ALs seperately.

### Group 1 (Short ALs)

The AL is the most important factor in IOL calculation. Any measurement error in the AL of a short eye could have a larger effect on final refractive error. Compression of the eye is believed to be part of the cause of AL shortening error [1–3, 17, 18]. A mean shortening of 0.25–0.33 mm has been reported between applanation and immersion axial length measurements, which can translate into an error of IOL power by approximately 1 D [3, 17–19].

There are different studies to evaluate the predictive accuracy of various IOL power calculation formulas in eyes with short AL by using different IOL calculation methods and different results are reported. Sander et al. [6, 16] and Narvaez et al. [20] reported that the SRK/T effective and no difference between any of third and fourth generation formulas at errors. Wang et al. [21] showed that the SRK/T and Hoffer Q were equal. Gavin and Hammond [17], Aristodemou et al. [12], Kapadia et al. [22], Hoffer Q [5], Szaflik et al. [23], Day et al. [24] showed that the Hoffer Q formula more accurate, contrary to Maclaren et al. [25], Terzi et al. [26], Moschos et al. [27] and Roh et al. [18] reported that the Haigis formula was more accurate than the other formulas.

In our study, the mean PE was 0.017 ± 0.58 D (range from −1.060 to 1.015) and there was a little tendency towards hyperopia. We found a prediction accuracy of 57.89 % for refractive errors of ±0.50 D, a prediction accuracy of 94.74 % for refractive errors of ±1.00D using SRK/T in eyes with short ALs (Table 2). The MAE of our study was 0.48 ± 0.29D (0.02 to 1.060). These results were similar to the previous studies or better than.

The ME was 0.87 D ± 0.829 D with SRK/T formula in 41 eyes with AL<22.00 mm using IOL master(IOLm) in the study conducted by Gavin and Hammond [17], 0.834 ± 0.262 D in eyes with AL<22.00 mm (*n* = 10; relatively small size series) by Hoffer et al. [5], 0.53 ± 0.25 D in 25 eyes with AL< 22.00 mm using IOLm by Roh et al.[18], 0.78 ± 0.66 D in 33 eyes with AL<22.00 mm using optical biometry by Wang et al. [21], 0.91 ± 0.64 D in 163 eyes with AL <22.00 mm using IOLm by Day et al., and 0.41 ± 0.23 D by Moschos et al. [27]. Contrary to the ME was −1.45 ± 0.14 D in 76 eyes with mean AL =20.79 mm by Maclaren et al. [25] and −0.96 ± −1.24 D in eyes with axial length < 21,00 D by Kapadia et al. [22]. Kapadia et al. reported that the postoperative SE within ± 1,0D was 80 % with SRK/T formula using A-Scan biometry.

In this study, we found a negative correlation between AL and K1, K2, IOLp; practically, as AL decreased, K1, K2,and IOLp increased. A negative significiant correlation was found between PEand and achieved SE, but no correlation between PE and the other parameters.

### Groups 2 (Long ALs)

The main difficulties in IOL power calculations for long eyes may be partly due to the anatomy of the posterior pole (posterior staphyloma). Posterior staphyloma decrease the accuracy of preoperative biometry. A-Scan biometry has a disadvantage compared with optic biometry and immersion biometry for accurate AL measurement [28–30]. Because of this, using A-Scan biometry with B-Scan mod together is recommended [29]. In our patients, sometimes A-Scan biometry was combined with B-Scan mod for detecting side of staphyloma.

The SRK/T formula probably the most accurate formula for long eyes and is now widely used. Holladay et al. [4], Sanders et al. [16],Hoffer Q [5], Kapadia et al. [22], Maclaren et al. [25], Donoso et al. [31], Kapamajian and Miller [32], Aristodemou et al. [12], El-Nafees et al. [33] and Chua et al. [34] were reported that SRK/T formula was more accurate than the other formulas in long eyes. Haigis et al. [15, 35], Terzi et al. [26], Bang et al. [36] and Roessler et al. [30] reported that the Haigis formula more accurate than the SRK/T formula and the others. Mitra et al. [37] and Petermeier et al. [38] reported that the SRK/T, Haigis or; Holliday were equal. Wang et al. [39] reported that the SRK/T and Haigis formula were comparable.

Sanders et al. [16] reported that for errors less than 0.5D was 45 %, less than 1.0D the results was 85 %, and greater than 2 D was 2.5 % by the SRK/T formula. In that study, there was no difference between SRK/T formula and the others. In the study conducted by Petermeier et al. [38], postoperative SE was −1.42 ± 1.33D (−3.94 to +1.0) (positive dioptre IOL group (*n* = 30) and postoperative SE was within ±0.5 D in 45.5 % of cases, and within ±1.0 D in 77.3 % of cases. Zaldivar et al. [29] reported that 92%of eyes were within ± 1.0D when using SRK/T formula in cases of plus power IOLs, and 54 % with the SRK/T in the cases of minus power IOLs. Maclaren et al. [25] did a retrospective analysis in 75 eyes having cataract surgery with zero- or negative-powered IOLs using SRK/T formula and A-scan, B-scan, and optic biometry. They also reported that forty-one percent of 75 patients analyzed were within ±1.00 D of the predicted refraction and 95 % confidence interval, 0.89-1.39 D. Kapadia et al. [22] reported that the MAE −0.59 ± 0.91D, −0.46 ± 0.24 D, 0.24 ± −0.05 D in eyes with axial length 24–27 mm (*n* = 28), 27–29 mm (*n* = 27), and >29 mm (*n* = 25) respectively, using SRK/T formula and A-Scan biometry. The postoperative SE was within ± 1 D in 67.85 % of cases when using SRK/T formula (Haigis equall; 68 %) in their study.

Ghanem and El-Sayed [28] reported that the postoperative SE was ± 1.0 D of assumed refraction in 75 %, the refractive outcome was within ±1.0D in 45 %, and there was a tendency toward hyperopia with SRK/T formula (*n* = 127, AL ≥ 26 mm). In the study conducted by Holladay et al. [4, 7], the ME was −0.194 D, and the MAE was 0.345 ± 0.401 D in eyes with AL 24.5–26.0 mm, the ME was 0.041, the MAE was 0.442 ± 0.56 D in eyes with AL greater than 26.0, and the MAE was 0.38 ± 0.47 D in all long eye. In a study consisting of more than 300 long eyes, Aristodemou et al. [12] reported that the SRK/T had the lowest MAE, with statistically significant differences for ALs of 27.00 mm or longer. Mitra et al. [37] found the ME was +0.92 D with SRK/T formula using applanation ultrasonography in Indian myopic population with long axial lengths (24.75–32.35 mm). Wang et al. [39] reported that the MAE was 0.45 ± 0.10 D with the SRK formula in eyes with AL more than 26 mm (*n* = 75). Narvaez et al. [20] reported the MAE was 0.49 ± 0.39 (0.00 ± 2.26 D; 24.5–26.0 mm), the MAE was 0.55 ± 0.64 D (range 0.04 ± 3.48 D; greater than 26 mm) in totally 181 eyes. El Nafees et al. [33] reported that the ME was +0.04 D (25–27 mm), +0.15D (27–29 mm), +0.33D (29–31.4 mm) with SRK/T and the MAE was less than 1.0D in 81.3 % eyes (*n* = 53 eyes). Chua et al. [34] reported that the ME was 0.18 D for eyes using SRK/T with ALs greater than 25 mm, Kapamajian and Miller [32] reported the mean PE was +1.16D and Roessler et al. [30] reported the MAE was 1.01 ± 0.61D with the SRK/T formula using optical biometry in long eyes.

In our study, the mean AL was 25.77 D (range from 24.60 to 32.90) and the mean IOLp was 15.80 (range from −5.00 to 20.50D). Only one patient had a negative IOL power. The postoperative SE was 0.42 ± 0.96 D (range; −2.62 to 2.75 D), within ± 1 D in 70.97 % of cases and within ± 0.5 D in 50 % of cases. The mean PE of in long ALs was −0.004 ± 0.93 D (range from −1.83 D to 3.55D) and there was a little tendency towards myopia. The MAE was 0.68 ± 0.62D (range 0.005 to 3.55D). We showed a prediction accuracy of 50 % for refractive errors of ±0.50 D, a prediction accuracy of 70.97 % for refractive errors of ±1.00D using SRK/T formula in eyes with long ALs (Table 2). We found a positive significant relationship between attempted preoperative SE and achieved postoperative SE (*r* = 0.255; *p* = 0.045). These results showed that refractive outcomes similar to the preoperative target refractive prediction ± 1D were reached. Only a few refractive surprises may be due to the AL errors in ultrasonic biometry or use of inappropriate formula.

Additionaly, the other results of our study can be summarized as follows:

1. We found no significant relation-ship between PE and AL,K1,K2, IOLp, IOL types, age in both groups.
2. We found a significant negative correlation for short ALs, contrary to a significant positive correlation for long ALs, between PE and achieved SE.
3. We found no significant relation-ship was found between MAE and AL, K1, K2, age, attempted SE, achieved SE in both groups.
4. We found a significant negative correlation between AL and K1,K2, IOLp in short ALs, a significant positive correlation between AL and K1,K2, contrary to negative AL and IOLp in long ALs.

This study has some weakness. Firstly, it is a retrospective analysis. Secondly, the relatively sample size (38 eyes) and a narrow range (20.50–21.99 D) of AL for the short eyes. Thirdly, only one formula(SRK/T) was used for IOL calculation and not compared with other formulas. Finally, different IOL types and IOL constants were used. On the other hand, our study also has some strength. Firstly, all surgeries and procedures were performed by a single surgeon with the same technique and devices. Secondly, relatively large sample size for long eyes (*n* = 62). Finally, using only one formula, the SRK/T formula, is an advantage of this study so as to determine the performance of a single formula.

## Conclusion

The results of the present study indicate that the SRK/T formula works well accurately in eyes with short and long ALs and shows a little tendency towards hyperopia for short, and myopia for long ALs. Further studies are needed to evaluate the performance of SRK/T formula in a wider range of eyes for short and long ALs. Additionally, this study suggests that the unexpected or unpredicted refractive outcome may happen. For this reason, ultrasonic biometry should be done carefully by an expert.

## Abbreviations

AL(s), axial length(s); IOL, intraocular lens; IOLm, IOL master; IOLp, intraocular lens power; K, keratometry value; MAE, mean absolute error; ME, mean (prediction) error; PCI, partial coherence interferometer; PE, prediction error; SD, standard deviation; SE, spherical equivalent; US, ultrasound
